# Advances in Measurement and Simulation Methods of Thin Liquid Film Corrosion

**DOI:** 10.3390/ma18194479

**Published:** 2025-09-25

**Authors:** Yikun Cai, Yuan Gao, Yixuan Zhuang, Shuai Wu, Fangyu Chen, Yiming Jin, Pengrui Zhu, Li Qin, Yan Su

**Affiliations:** 1 School of Aeronautics and Astronautics, Sichuan University, Chengdu 610207, China; caiyikun@scu.edu.cn (Y.C.);; 2 Southwest Technology and Engineering Research Institute, Chongqin 400039, China

**Keywords:** thin liquid film, thickness measurement, electrochemical property, corrosion simulation

## Abstract

Thin liquid film corrosion is a critical failure mechanism for the atmospheric environment and industrial infrastructure. This review systematically examines relevant methods and recent advances in characterizing and simulating this phenomenon. Various measurement methods for liquid film thickness, composition, and conductivity are investigated, with particular focus on the advantages of non-contact optical technology and X-ray fluorescence (XRF) in in situ monitoring and analysis. For corrosion simulation, the finite element method (FEM), cellular automaton (CA), and molecular dynamics (MD) are widely used. Their combination has synergistic potential in revealing essential corrosion mechanisms and establishing prediction models across scales.

## 1. Introduction

Thin liquid film corrosion is a major cause of failure for marine equipment, pipelines, and other facilities, as metallic materials are often exposed to the humid or condensation conditions [1]. As the corrosion damage accumulates over time, material performance deteriorates, leading to structural failure and potential catastrophic accidents. The atmospheric corrosion of metals is fundamentally an electrochemical reaction process under liquid film coverage, which acts as the medium for mass transport (e.g., ions, oxygen, corrosive gases) and electrochemical reactions of the corrosion process. The physicochemical properties of the liquid film, such as thickness distribution, ion concentration, electrochemical parameters, are the controlling factors of the corrosion kinetics. However, thin liquid film exhibits non-continuous, heterogeneous, and dynamic distributions on metal surfaces, and is influenced significantly by humidity, temperature, airflow, and other factors. Thus, it is challenging to describe its spatial distribution and dynamic evolution patterns and to quantify its corrosive effects.

From the mechanistic perspective, the corrosion rate is controlled by mass transport within the liquid film and electrochemical reactions at the electrode surface. Physical models describing this process involve coupled partial differential equations. However, it is often hard to obtain analytical solutions due to the complexity of film morphology and nonlinear boundary conditions [2]. The development of numerical simulation technology offers new pathways to overcome these challenges. The accuracy relies on precise modeling of liquid film properties, including spatiotemporal thickness distribution, ion migration characteristics, interfacial charge transfer, and other parameters.

Two critical challenges remain in thin liquid film thickness measurement and numerical corrosion simulation: (1) Dynamic film evolution (i.e., condensation, evaporation, flow) leads to spatial and temporal differences in physicochemical properties, which requires non-contact measurement and continuous monitoring at the global scale of the whole metal surface. (2) The vast spatiotemporal gap (six orders of magnitude) should be bridged between microscopic mechanisms (molecular dynamics simulations at micrometer–microsecond scales) and macroscopic predictions (engineering lifespan assessment at meter-year scales).

Regarding the above issues, this review systematically summarized recent advances in liquid film corrosion in three aspects: (1) thickness measurement, (2) physical and chemical properties measurement, and (3) numerical simulation methods. This can help to explore the essential mechanisms of thin liquid film corrosion and establish accurate corrosion prediction models.

## 2. Measurement of Thin Liquid Film Thickness

**Figure 1:**
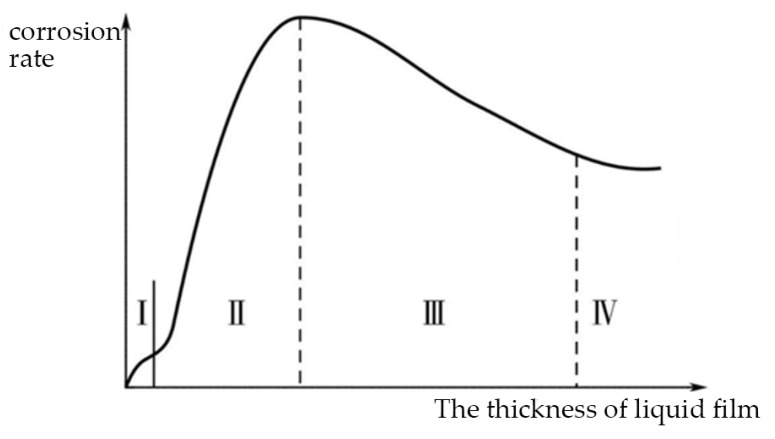
Relationship between the corrosion rate and the thickness of the liquid film.

The thickness of thin liquid film is the most basic and important parameter that influences the corrosion rate. Figure 1 shows that when the film thickness is below a lower limit (Zone I), the corrosion rate remains unchanged or decreases as the film thickness decreases. As the film thickness gradually increases (Zone II), the corrosion rate will significantly increase and reach its maximum value. When the thickness of the liquid film increases again (Zone III), the corrosion rate will gradually decrease and reaches a stable value (Zone IV). This trend has been proved by various studies [3,4]. However, for different metals serving in different environments, there have been no consistent values for the ranges of the four zones and the corrosion rate. Therefore, it is of great significance to study the thickness of liquid film for establishing the corrosion kinetics model. This paper screens the current thickness measurement methods and summarizes their advantages and disadvantages in a complex environment.

### 2.1. Electrical Measurement Method

Electrical measurement methods mainly include two types: (1) determining the liquid film thickness through the signal difference (current and voltage) between the first contact with the liquid film and the arrival at the metal; and (2) detecting the electrical parameters (capacitance and conductance) of the liquid film and transforming it into thickness values. There are three most commonly used methods: the probe method, the capacitance method, and conductance method.

**Figure 2:**
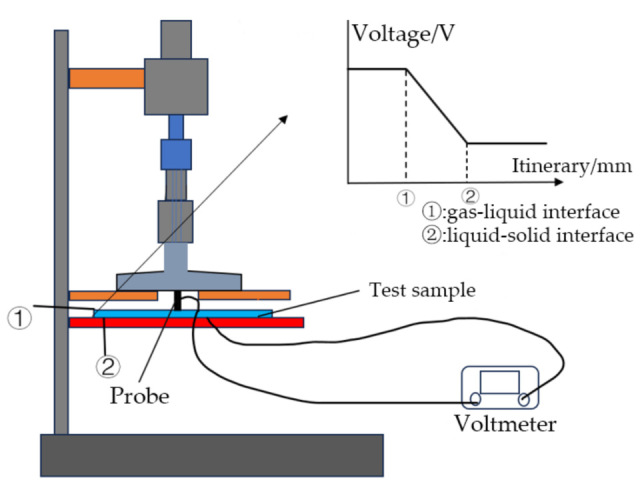
A probe–type liquid film thickness measuring device.

The probe method is intuitive. As shown in Figure 2, when the probe contacts with the gas–liquid interface, the voltage undergoes a sudden change due to the formation of a current path, and the displacement at this moment is recorded as $d_{1}$. When the probe touches the solid–liquid interface, the voltage changes abruptly again because of the sudden change in electrical conductivity, and the displacement at this moment is recorded as $d_{2}$. The displacement difference $d_{2}-d_{1}$ is the thickness of the liquid film [5,6].

Jeon-Hong Kang [7] improved the probe contact measurement and developed a four-point probe thickness gauge for conductive films, which is used to measure the film thickness during the manufacturing process of flat panel displays such as touch screens.

**Figure 3:**
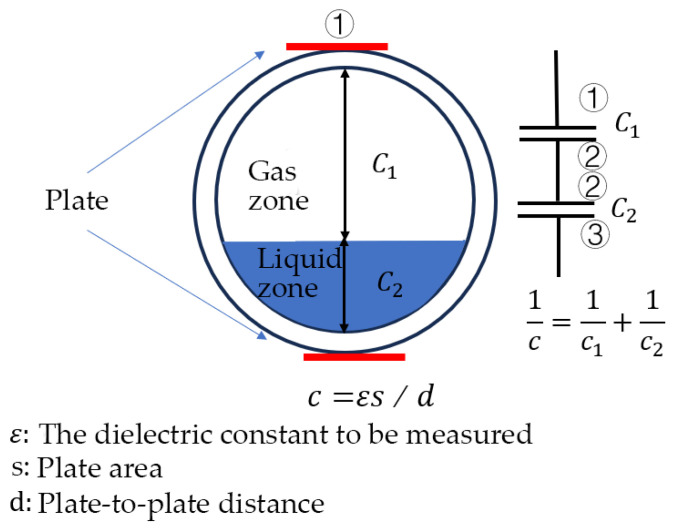
A device for measuring liquid thickness by capacitance.

The capacitance contact measurement method is based on the analysis of the capacitor formed by the electrodes arranged on both sides of the flow channel. As shown in Figure 3, when the liquid height around the wire changes, the capacitance of the instrument also changes [8].

According to the capacitance formula for parallel plates, $C=(\varepsilon_{0}\cdot \varepsilon_{r}\cdot s)/d$; when $\varepsilon_{0}$, $\varepsilon_{r}$, *s* are constant, the capacitance *C* is inversely proportional to the thickness(*d*) of the liquid layer.

Generally, since the dielectric constant of liquid is much higher than that of air, the capacitance of air can be neglected. However, water vapor in the air has a significant impact on the air capacitance. To reduce measurement errors, Hussein [9] improved the classic configuration of the capacitance contact method. They employed a three-capacitor approach to eliminate errors caused by air capacitance and improve measurement accuracy.

The measurement process of the electrode contact method can be shown as below. The liquid film between two parallel conductive electrodes can be regarded as a conductive medium layer, and its resistance (*R*) and the liquid film thickness (*h*) satisfy the following relationship: R=h/(σ·s), where *S* is the contact area between the electrode and the liquid film, and σ is the electrical conductivity of the liquid, which needs to be known in advance or obtained through calibration. This equation indicates that when the electrical conductivity and electrode area are fixed, the film thickness is proportional to the measured resistance. Thus, the liquid film thickness can be measured by determining the resistance value. Based on this method, Wang Chao [10] used a conductivity probe to directly measure the thickness of wavy liquid films in a gas–liquid two-phase annular flow with reduced measurement errors.

Electrical measurement methods have high temporal resolution, require less sophisticated instruments, involve straightforward data processing, and only need simple operation. They can utilize parallel dot matrix measurement techniques to simultaneously measure the thickness of liquid films at multiple points.

However, as these methods are all contact measurement, there may be some challenges when they are applied to atmospheric thin liquid corrosion: (1) The original form of the liquid film will be destroyed during the measurement; (2) the electrical properties of liquid film with different components should be measured in advance; (3) non-conductive liquid film cannot be measured; and (4) the spatial difference of the liquid film cannot be detected.

### 2.2. Microwave Resonance Method

When the microwave is radiated from the probe into the multi-layer structure, the reflected and transmitted signals at each boundary will produce an overall reflected signal. The signal contains information about the complex dielectric characteristics and thickness of each layer [11].

As the dielectric constants of air and liquid film are significantly different, the resonant frequency *f* and energy loss *Q* are also different in the resonant cavity. By measuring the resonant frequency shift Δf and the energy loss change ΔQ, the film thickness *d* can be calculated by the standard Δf−d (or ΔQ−d) curve.

Based on this method, Gabriel Galindo-Romera [12] designed a sensor with a split-ring resonator. By measuring the offset frequency of the resonant cavity, this sensor realizes the non-contact and rapid measurement of different types of liquid films with thicknesses ranging from 100 μm to 1 mm. It can also be used to detect solid thickness and relative permittivity characteristics.

This microwave resonance method has high reliability. However, the measurement accuracy, which is about the order of 100 μm, is limited. When the liquid film is less than this value, the measurement accuracy cannot be guaranteed.

### 2.3. Ultrasonic Method

**Figure 4:**
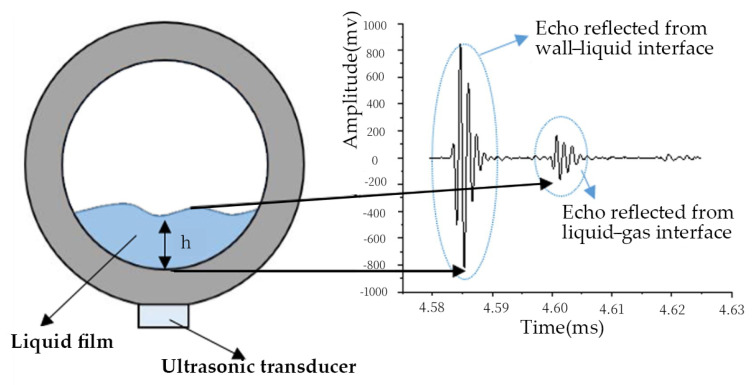
Principle of ultrasonic method to measure film’s thickness.

When ultrasonic waves propagate through multi-layer media, the interfaces cause acoustic impedance non-continuities, leading to the reflection of the waves. As shown in Figure 4, echoes are generated at specific interfaces, and the liquid film thickness can be calculated through the sound velocity and time difference between two reflected signals. Al-Aufi, Y.A. [13] utilized this method to investigate wavy liquid films (<6 mm) in vertical pipes. Different signal processing methods are proposed to reduce the measurement error in the millimeter range.

The ultrasonic method responds quickly and is particularly suitable for flowing liquid measurement. However, its accuracy is limited by the wave length (in the order of millimeter). Moreover, as the acoustic impedance difference at the interface is very large, nearly total reflection happens at the solid–liquid interface. The echo reflected from the liquid–gas interface is relatively weak.

### 2.4. Optical Method

#### 2.4.1. Polarization Method

Typically, when linearly polarized light with a known polarization state is incident on the surface of a sample, the polarization state of the reflected (or transmitted) light will change to elliptically polarized light, which consists of the p-polarization component and the s-polarization component. The polarization method is mainly based on the Fresnel equations, which correlate film thickness *d* with polarization parameters Ψ and Δ. Ψ presents the reflection coefficients difference between p-polarized and s-polarized light, while Δ is the phase difference. By measuring Ψ and Δ, the thickness of films can be determined. Based on this method, Yuan Yucong [14] investigated atomic-scale film thickness using a liquid crystal variable retarder (LCVR) and achieves high angular resolution.

The polarization method is non-contact and has very high accuracy (sub-nanometer). It is also suitable for non-transparent samples and needs no reference before measurement. But its measurement range is relatively narrow. It is not suitable for the measurement of liquid film thickness on metal surfaces with large thickness (micrometer).

#### 2.4.2. Interferometry

(1) Monochromatic light

**Figure 5:**
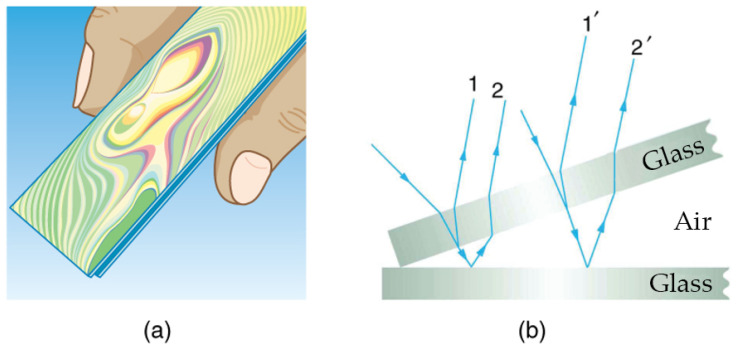
Thin-film interference: (**a**) thin-film interference fringe pattern, and (**b**) principle diagram of thin-film interference.

Monochromatic light interference is based on the principle of thin film interference. As shown in Figure 5, reflection and refraction at the film interface create an optical path difference between light rays. This path difference turns into a phase difference, leading to constructive or destructive interference. Due to the different degrees of interference at different positions, alternating bright and dark interference fringes will appear (white light will produce colored fringes). The spacing of these bright and dark fringes contains information related to the thickness of the thin film. The thickness of the liquid film can be calculated according to the interference formulas, such as 2n·d·cosθ=k·λ or 2n·d·cosθ=(k+1/2)·λ, where *n* is the refractive index of the liquid film, *d* is the liquid film thickness, θ is the refraction angle of light in the liquid film, *k* is the interference order, and λ is the wavelength of light. Monochromatic light interference typically produces high-contrast fringe patterns, making it suitable for wide-range film thickness measurements [15]. The excellent coherence of monochromatic light further enables high-resolution imaging of thickness variation fields [16]. Elizaveta Y. Gatapova [17] used this method to study liquid film evaporation, achieving 3D thickness reconstruction and tracking for films under 40 nm while quantifying evaporation rates.

(2) White light

**Figure 6:**
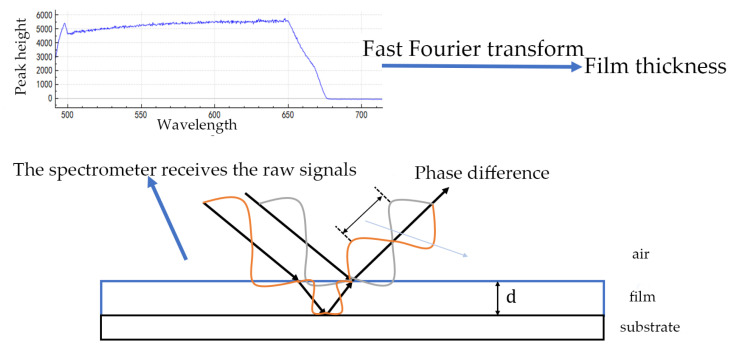
Basic principles and steps of white light interferometry for thickness measurement.

White light exhibits low coherence. As shown in Figure 6, When incident on a “substrate–liquid film–air” structure at an angle, the light behaves in two ways. First, a portion is reflected at the upper surface of the liquid film (the liquid–air interface). Then, another portion travels through the film and is reflected at the lower surface (the substrate–liquid interface). The two reflected light beams produce an optical path difference due to their different propagation paths. Since white light contains light of various wavelengths, only when the optical path difference of the two reflected light beams (related to the liquid film thickness, refractive index, and incident angle) is an integer multiple of a certain wavelength will the light of that wavelength undergo constructive interference, forming a bright fringe corresponding to that wavelength, while destructive interference occurs when the optical path difference is a half-integer multiple of the wavelength.

The system is brought to the zero optical path difference position by scanning the film thickness or adjusting the optical path. Here, the optical path difference is zero, which causes all wavelengths to interfere constructively at once and form a high-contrast white central fringe. Accurate calculation of the liquid film thickness is achieved by analyzing the relationship between this zero optical path difference position and the optical paths of the reflections from both film surfaces, along with the film’s refractive index and the incident light parameters.

From the perspective of specific scanning methods, white light interferometry can be divided into two types: (1) Z-scan uses a mechanical scanner to change the optical path difference between the measuring arm and the reference arm while monitoring the generated interferogram; and (2) K-scan involves gradually scanning the wavelength of white light using a tunable laser acousto-optic modulator and a liquid crystal cavity. Compared with Z-scan, K-scan has the advantage of rapid measurement without a mechanical scanner [18]. Guo [19] proposed two initial estimation methods based on nonlinear phase frequency and overall visibility of the spectral signal.

The monochromatic light interference spectroscopy method has high measurement accuracy and it is not easy for it to be disturbed. The white light interference spectroscopy method not only has the aforementioned advantages but also features fast response speed, strong stable ability, and abundant information contained in the signal. However, its measurement range is narrow, and there is phase ambiguity when the phase difference is greater than 2π.

#### 2.4.3. Total Internal Reflection Method

**Figure 7:**
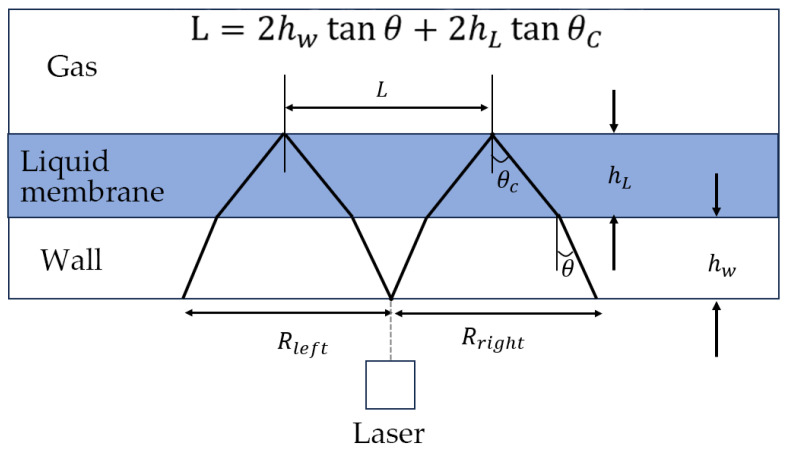
Principle of thickness measurement by total internal reflection method.

When light transmits from light dense medium to light sparse medium, total reflection will occur if the incident angle is greater than the critical angle. Figure 7 shows the position where the total reflection occurs. It can be seen that the intensity of the reflected light and the brightness of the spot formed on the wall are low before the total reflection occurs. When total reflection occurs, the brightness of the light spot on the bottom surface will increase significantly, and an aperture with high brightness is formed. According to the position of the aperture, the thickness of the liquid film can be calculated. Matteo Grasso conducted an error analysis on the measurement of thin film thickness using the total internal reflection method. The analysis includes the potential impact of refractive index changes on the measured thickness, the extension of the experimental calibration range to a wider measurable thickness, and the influence of unevenness of the film-free surface with the measured thickness [20].

The total internal reflection method has high measurement accuracy and requires simple instruments. However, it needs a transparent substrate, so its applicable scenarios are limited.

### 2.5. Fluorescence Intensity Method

Fluorescent substance (fluorophore) is a compound that can emit and absorb external radiation repeatedly. The solid or liquid fluorophore is dissolved in the working liquid, and the working liquid is irradiated by a laser with a wavelength close to the wavelength of the maximum light absorbed by the fluorophore [21]. According to the fluorescent oil flow pictures obtained by the camera, different concentrations of fluorescent molecules reflect different regional brightness (the thickness of the fluorescent liquid film). Thus, we can roughly establish a relationship between the brightness and the thickness of the fluorescent liquid film with the formula h=k·l, where *h* is the thickness, *l* is the brightness, and *k* is the scale factor. The thicker the fluorescent liquid film, the higher the brightness of the corresponding area. The thickness can be obtained by reading the brightness [22].

This method has a high signal-to-noise ratio and is convenient for signal processing. However, adding fluorescent indicators will change the original components of the liquid film and interfere with the atmospheric corrosion of the metal under the liquid film.

### 2.6. Current Challenges and Opportunities

Different liquid film thickness measurement methods have their own advantages and limitations as summarized in Table 1. Electrical measurement techniques, despite 1 ms level time resolution, suffer from >100 μm spatial resolution and a 30% accuracy drop for non-conductive liquids. Microwave resonance methods, ideal for dynamic monitoring, are confined to about 100 μm precision due to wavelength constraints. The ultrasonic method (10 μm precision) faces a low signal-to-noise ratio at the air–liquid interface and is sensitive to the environment. Optical methods have high measurement precision (about 0.1 μm) while suffering from complex calibration (300 s calibration for white light interferometry) or a narrow measurement range (<1 μm range for ellipsometry). Fluorescence techniques achieve a high signal-to-noise ratio and global measurement, but the added fluorescent agent will change the compositions and properties of the liquid film.

In atmospheric corrosion, the focus is on the electrochemical behavior within the thickness range of 1–100 μm. As the liquid film is non-uniform, non-continuous, and varies along with time, the thickness measurement methods should be global, non-contact, and in situ, and capable of covering the entire range of liquid film thickness with high precision. In view of this, the optical method is the most promising option. Its combination with other methods may also be a good way to leverage the advantages of different methods.

## 3. Measurement of Thin Liquid Film Composition and Electrochemical Parameters

As core parameters characterizing the chemical environment of thin liquid films, chloride ion concentration, pH, and electrical conductivity carry critical information about the film’s composition, ionic migration capability, and acid–base equilibrium state. Chloride ions, due to their strong penetration ability and depolarization effect, are the primary aggressive ions inducing metal corrosion. Electrical conductivity reflects the total ion concentration and migration rate within the liquid film, directly influencing the current intensity of corrosion cells. pH value, by regulating the stability of the passive film on the metal surface (such as the balance between anodic dissolution and cathodic hydrogen evolution reactions), determines the transition of corrosion mechanisms (e.g., uniform corrosion versus localized corrosion). Researchers have developed various measurement techniques focused on the physicochemical properties of thin liquid films. Significant progress has been made in measuring chloride ion concentration, pH value, and electrical conductivity.

### 3.1. Chloride Ion Concentration

#### 3.1.1. Mohr Titration Method

The fundamental principle of the Mohr method for determining chloride ion concentration involves the reaction of chloride ions with a standard solution of silver nitrate or mercury nitrate to form a precipitate. The titration endpoint is determined using an indicator. By measuring the volume of the standard solution consumed, the chloride ion content is finally calculated. The chemical reaction equation is as follows:

For silver nitrate titration:

(1) $$Cl^{-}+Ag^{+}\to AgCl↓$$

For mercury nitrate titration:

(2) $$2Cl^{-}+Hg^{+}\to HgCl_{2}↓$$

However, the Mohr method has exposed many problems due to its rudimentary procedures. Scientists have gradually resolved some of these issues through various means. Yokoi [23] successfully used mercury nitrate titration to determine chloride ion content in rat urine. Although achieving high sensitivity, the method was only suitable for measuring small sample volumes. Shivaji R. Labhade [24] successfully synthesized a mercury(II) thiocyanate chloride compound [Hg(SCN)Cl] for determining chloride in beer by mercury nitrate titration. This compound was simple to prepare and maintained the homogeneity of the reaction medium under acidic conditions, making it suitable for the rapid determination of chloride in beer.

Although the Mohr method was proposed over 100 years ago, it suffers from issues such as low recovery rates as the test solution readily reacts with the titrant. Furthermore, for measuring the composition of liquid films in atmospheric corrosion, it is difficult to collect the test solution from the metal surface before measurement as the liquid film is rather thin. This non-in situ measurement approach limits its application in atmospheric corrosion research.

#### 3.1.2. Ion-Selective Electrode (ISE) Method

**Figure 8:**
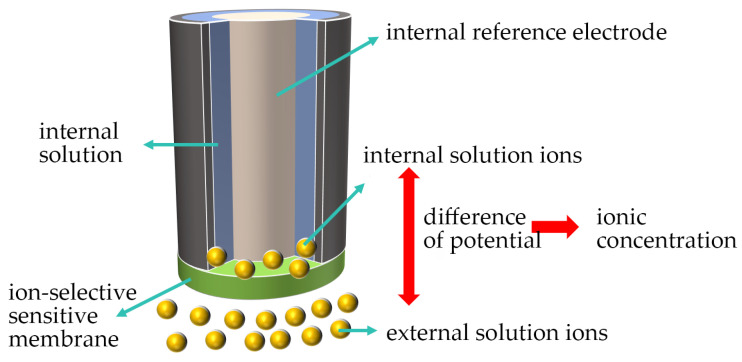
Principle of ion-selective electrode.

An ion-selective electrode (ISE) is an electrochemical sensor whose core component is a sensitive membrane with high perm-selectivity for specific ions (e.g., chloride ions). As shown in Figure 8, when the electrode is immersed in the test solution, the difference in chloride ion concentration across the sensitive membrane generates a potential difference. This potential difference is proportional to the logarithm of the chloride ion activity. By measuring this potential difference and combining it with the known electrode response characteristics, the chloride ion concentration in the solution can be calculated.

The ion-selective electrode method generally employs two types of systems, two-electrode and three-electrode systems, as shown in the figure. Ping [25] developed a chloride-selective solvent polymeric membrane electrode based on ionophore that forms hydrogen bonds. This electrode significantly reduced the interference from ions such as sulfite ion, bromide ion, and bicarbonate ion. It provides a new tool for clinical analysis and environmental monitoring, demonstrating the potential for chloride determination in complex samples. Pietrzak [26] developed a solid-contact chloride ion-selective electrode based on a composite material of polyaniline nanofibers and multi-walled carbon nanotubes. Compared to traditional chloride electrodes, this electrode offers advantages such as a wider measurement range, better potential stability, and a lower detection limit.

In the treatment of thin liquid films, Liu, Y. [27] studied the ion concentration measurement of thin-layer samples with an average coulometry efficiency of 96%.

However, the ion-selective electrode method requires inserting the probe into the solution for measurement, which may disrupt the surface distribution of the liquid film and affect the measurement results. Additionally, if the liquid film is thin, large-sized probes may be unable to measure the chloride ion concentration. Therefore, current ion-selective electrodes are often embedded into smaller electrodes and the bonding of the substrate and electrode.

#### 3.1.3. Microwave Detection Method

The microwave detection method achieves concentration detection by analyzing changes in the characteristic parameters (such as the dielectric constant) of microwaves before and after transmission through the test solution. A clear relationship exists between the solution concentration and the dielectric constant, with which quantitative detection of the solution concentration can be achieved [28].

The microwave detection method can be specifically subdivided into various forms based on the measurement approach of microwaves towards the sample, including the resonance method, reflection method, and so on.

Among these, the principle of the resonance method is that microwaves generated by a microwave emitter form a standing wave field within a resonant cavity, using a resonant circuit or device with a high quality factor as the sensing element. When the test solution is introduced into the resonant cavity, changes in its dielectric properties alter the resonant frequency and the quality factor. By detecting the shifts in these two parameters, a quantitative relationship with solution concentration can be established.

The measurement principle of the reflection method is that when microwaves propagate from a sensor towards the test solution, changes in the solution’s dielectric properties cause an abrupt change in the impedance of the propagation medium, leading to partial reflection of the incident wave. A microwave vector network analyzer is used to simultaneously detect the phase information of the incident and reflected waves, and the ratio of the two is taken as the reflection coefficient. By experimentally calibrating the characteristic reflection coefficient values corresponding to solutions of different concentrations, a mathematical model for concentration detection can be constructed.

**Figure 9:**
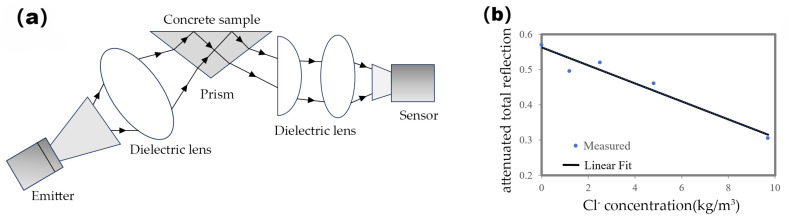
(**a**) The principle of the microwave attenuation total reflection measurement device. (**b**) The attenuation total reflection–chloride ion concentration graph [30].

Based on the principle of the reflection method, Tripathi [29] designed a microwave attenuated total reflection (ATR) measurement setup, with a schematic diagram shown in Figure 9. Using this method, they observed that the reflectance near 65 GHz decreased almost linearly with increasing chloride ion concentration, with a correlation coefficient reaching 0.97. This enabled the non-destructive detection of chloride ion concentration within the range of 0–10 mg/cm^3^ in concrete structures.

All the chloride ion concentration measurement methods based on microwave detection mentioned above require no sample preparation and enable in situ measurement. However, this method often requires establishing specific calibration curves for samples with particular compositions and is typically used in measurement scenarios where the composition is simple or relatively fixed. It is difficult to achieve widespread application in practical complex scenarios where the composition of the medium fluctuates significantly.

#### 3.1.4. X-Ray Fluorescence

X-ray fluorescence (XRF) is a non-destructive, high-precision analytical technique for elemental composition and content measurement [31]. When high-energy X-rays (with energies higher than the binding energy of inner atomic electrons) strike an atom, an inner-shell electron is ejected and a vacancy is created. The atom is in an unstable excited state and will spontaneously transition from high-energy to low-energy configuration. The emitted X-ray energy precisely equals the energy difference between the two electron orbitals involved.

**Figure 10:**
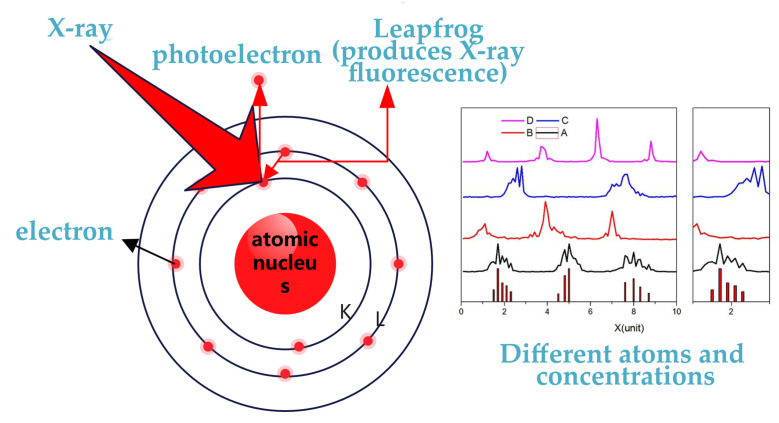
Principle of X-ray fluorescence analysis.

As shown in Figure 10, since each element possesses a unique atomic energy structure, the X-ray fluorescence emitted during excitation and transition exhibits element-specific energy characteristics. These emissions are called characteristic X-rays. A mathematical relationship exists between an element’s atomic number and its fluorescent X-ray wavelength. This allows elemental identification through wavelength measurement. Concurrently, the fluorescence intensity depends on how many atoms are excited, enabling quantitative analysis by establishing a relationship between intensity and elemental content.

Qualitative analysis is often based on Moseley’s law: $\sqrt{1/\lambda }=k\cdot \left(Z-\sigma \right)$, where, λ is the characteristic X-ray wavelength, *Z* is the atomic number, and *K* and σ are constant in regard to the electron transition level. By measuring the wavelength spectrum emitted by the sample and comparing it with the element standard spectrum library, the element species can be obtained. For quantitative analysis, attention should be paid to the relationship between element content and X-ray intensity $I_{i}=k\cdot C_{i}\cdot S_{i}$, where $C_{i}$ is the element content, $I_{i}$ is the X-ray intensity, $S_{i}$ is the matrix correction factor, and *k* is the instrument constant.

XRF analysis is a non-destructive and non-contact technology, making it ideal for scenarios where traditional analytical methods are limited. The X-ray fluorescence analyzer is capable of rapid measurement and the fluorescence spectra are unaffected by molecular bonding states in test samples. These instruments can provide high resolution and in situ measurements. However, as the instrument performance and longevity are significantly affected by temperature, humidity, and other external factors, most commercial XRF spectrometers are primarily deployed in the laboratory [32].

### 3.2. PH Value

#### 3.2.1. Electrochemical Measurement Method Using Glass Electrode pH Meters

When a glass electrode is immersed in the solution under test, an electrical double layer forms on both sides of the glass membrane. Due to the selective response of the glass membrane to hydrogen ions, the concentration difference in $H^{+}$ across the membrane generates a membrane potential, which exhibits a linear relationship with the pH value of the solution. By measuring the electromotive force (EMF) of the electrochemical cell, the solution’s pH can be calculated using the Nernst equation. However, the immersion process itself may slightly perturb the solution, leading to measurement inaccuracies. Thomas, R.C. [33] developed a novel recessed-tip pH-sensitive glass microelectrode, which enables intracellular pH measurement by inserting only the extreme tip into cells. The specific values are shown in Table 2. This electrode was successfully applied to measure pH in snail neuron cells, demonstrating the broad application potential of novel pH-sensitive microelectrodes in cellular physiology research.

Andrea Salis [34] investigated the influence of electrolytes on pH measurements using glass electrodes based on the Hofmeister effect. The results indicated that the Hofmeister effect is closely related to physicochemical parameters of ions such as polarizability, partial molar volume, and surface tension increment. These parameters influence ion behavior in solution through non-electrostatic (NES) or dispersion forces, thereby affecting pH measurement values. This provides a further explanation for the errors generated in pH measurements with glass electrodes.

#### 3.2.2. Optical Measurement Method Based on Surface-Enhanced Raman Spectroscopy (SERS)

Raman spectroscopy is a scattering spectroscopy technique, the key to which lies in the inelastic scattering between light and molecules. When monochromatic light (e.g., laser light) irradiates a sample, most of the light undergoes Rayleigh scattering (with the same frequency as the incident light), while only approximately 0.001% of the light undergoes Raman scattering. The frequency shift in the Raman-scattered light corresponds to molecular vibrational or rotational energy level transitions, producing characteristic Raman shifts that reflect molecular structural information.

**Figure 11:**
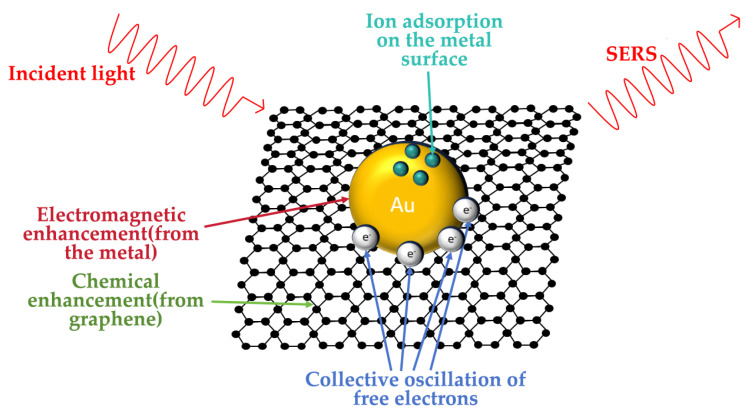
The principle of surface-enhanced Raman spectroscopy technology.

Subsequently, ions in the solution are adsorbed onto the SERS substrate surface through electrostatic interactions, coordination bonds, or specific binding, as shown in Figure 11. Changes in ion concentration leads to variations in the adsorption amount on the substrate surface, thereby altering the intensity of characteristic Raman peaks. By measuring the Raman peak intensities of solutions with known ion concentrations, a concentration-intensity calibration curve is established, enabling quantitative analysis of unknown concentrations. The core of surface-enhanced Raman scattering (SERS) technology lies in the significant enhancement of the Raman signal through metallic nanostructures. The enhancement mechanisms primarily include electromagnetic field enhancement and chemical enhancement.

Aleksandra Jaworska [30] conducted research on intracellular pH measurement using surface-enhanced Raman scattering (SERS) and fluorescence microscopy techniques. Kendrich O. Hatfield [35] achieved real-time monitoring of surface pH changes based on a novel surface-enhanced Raman spectroscopy-scanning electrochemical microscopy (SERS-SECM) technique, demonstrating high sensitivity. Cao [36] described several advanced studies aimed at showcasing the advantages of SERS as a versatile tool, not only for investigating aqueous interfaces but also for studying transition metals at non-aqueous interfaces.

Mancio, M. [37] employed electrochemical and in situ surface-enhanced Raman spectroscopy techniques to investigate the passive film characteristics and corrosion performance of 9% Cr micro-composite steel in highly alkaline environments. They explored the influence of pH and ionic strength on corrosion behavior.

The research results revealed the excellent corrosion resistance of the micro-composite steel in highly alkaline environments, particularly its ability to maintain polarization resistance without significant reduction upon pH decrease. This provides crucial evidence for the corrosion resistance of micro-composite steel in practical applications.

Although SERS is label-free and has high-precision, its complex preparation and high cost have currently limited its application primarily to the field of biological research. Due to issues with substrate stability in corrosive environments and interference from corrosion products, this technology has not yet been widely adopted in corrosion research.

### 3.3. Conductivity

The primary principle of electrical conductivity measurement involves applying an electric field or electromagnetic field excitation to the object under test and measuring its response. Then, the conductivity of the measured object is determined through analysis based on charge transport or electromagnetic field theories. The equivalent circuit is the most commonly used method. The measurement techniques have been developed for a long time. To reduce measurement errors, various excitation methods have been employed. For example, Wei [38] designed a conductivity instrument based on the MSP430F149 microcontroller and employed a bipolar pulse excitation signal to mitigate the influence of electrode polarization effects. The structure of the measurement system is different, mainly including the bridge method, the three-electrode method, and the four-electrode method.

The bridge method is to connect the bridge circuit to the solution to be tested. By adjusting the resistance value of the bridge arm, the bridge reaches equilibrium. Combined with the conductance cell constant, the conductivity of the solution can be calculated. Polgar, K. [39] measured the effect of magnesium doping on the electrical conductivity of LiNbO_3_ single crystals using the alternating current bridge method, revealing the mechanism of change in the crystal defect structure at a specific magnesium concentration. As the accuracy of conductivity measurement is affected by inherent capacitance and the spontaneous electromotive force of liquid, Liu [40] used a trapezoidal wave with a frequency of 1 kHz and an amplitude of 5V as the excitation signal to achieve the high-precision conductivity measurement of seawater using the improved HD-4 salinity meter. The improved salinity meter makes the measurement more accurate and rapid, and it also has an automated function.

The three-electrode conductance consists of one intermediate electrode and two terminal electrodes. The current flows into the solution to be tested from the intermediate electrode and then out from the two terminal electrodes. By measuring the voltage between the two terminal electrodes, the equivalent resistance (conductivity) of the solution can be calculated. Abdurahman Radjabov [41] developed a three-electrode system to study the electrical conductivity (electrical resistance) of the pulp and skin of fruits and grapes, which showed sufficient measurement accuracy. The three-electrode method focuses on controlling the potential of the working electrode and is suitable for the study the interfacial process in the solution.

The four-electrode conductance consists of two outer current electrodes and two inner voltage electrodes. An AC current is injected via the outer pair and the resulting voltage drop is measured across the inner pair. Zhao [42] proposed a simple control and high-accuracy measurement method for a four-electrode conductivity probe using bi-directional voltage pulse. The relative error of the conductivity measurement between the laboratory and the field results is within 2.5% in a conductance range of 10 μSm/cm to 200 mS/cm. The four-electrode method significantly reduces errors arising from contact resistance and electrode polarization due to its unique configuration. Consequently, it is widely used in conductivity measurement across diverse fields.

There are also noncontact conductivity sensors, in which there is no galvanic contact between the electrodes and the solution. Two types are the most commonly used: capacitive coupling and inductive coupling [43]. Their application to thin liquid film measurement and atmospheric corrosion needs further study.

### 3.4. Current Challenges and Opportunities

Mohr titration offers a straightforward approach for determining Cl^−^ concentration, but it is not suitable for in situ applications. In contrast, the ion-selective electrode method enables in situ, high-precision measurement via the potential response. Since this method is contact-based and has a relatively large size, it cannot be used within micron-scale liquid films. For non-contact alternatives, the microwave method correlates dielectric constant changes with Cl^−^ concentration and enables rapid field detecting, but it will be interfered with by organic compounds. X-ray fluorescence (XRF) excites characteristic X-rays to measure Cl^−^ concentration at interfaces. However, its weak anti-interference ability requires a stable laboratory environment. Future Cl^−^ monitoring requires non-contact techniques with higher spatial resolution and enhanced resistance to interference within micro-scale environments.

The pH of liquid films plays a critical role in determining passivation stability, corrosion kinetics, and inhibitor effectiveness. Glass electrode pH meters are well established and widely used in industrial applications due to their rapid response and operational simplicity. However, they are contact-based and need frequent calibration to avoid ionic interference. In contrast, surface-enhanced Raman spectroscopy (SERS) utilizes plasmonic nanostructures to provide ultra-high sensitivity and non-destructive molecular characterization, showing great promise for localized corrosion analysis. The main challenges of SERS include complex substrate fabrication and high cost. Future pH sensing requires non-contact techniques with high precision and broad applicability in harsh or confined electrochemical environments, such as simplified and robust SERS platforms.

Conductivity reflects total ionic concentration and mobility, and governs local ion transport and corrosion rates. The classical bridge method is stable and precise via balanced circuit measurements but is operationally complex and slow. The three-electrode (electrochemical cell) method facilitates potential control and interfacial parameter extraction during mechanistic studies, yet cannot directly measure bulk conductivity and requires complex instrumentation. The widely adopted four-electrode method employs separate pairs for current injection and voltage sensing, allowing direct measurement of bulk conductivity with minimal polarization error. However, this approach requires calibration using known geometric constants and cannot offer insight into interfacial properties. Future conductivity measurement seeks techniques combining bulk and interfacial analysis capabilities with simplified calibration and real-time monitoring.

Their advantages and limitations are classified and explained in Table 3. In summary, while existing techniques for monitoring Cl^−^, pH, and conductivity each provide unique strengths, they still face three major challenges: (1) achieving non-contact, in situ detection with high spatial resolution within thin liquid films and interfacial regions; (2) minimizing interference and lowering system complexity and cost; and (3) developing integrated platforms to enable simultaneous and multi-parameter analysis.

## 4. Corrosion Simulation Methods

**Figure 12:**
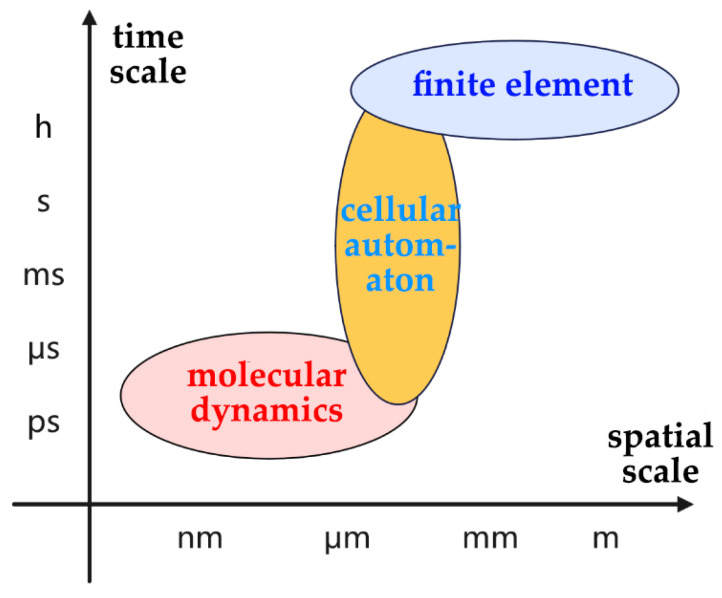
Time-spatial scale of simulation methods.

Based on the collected physicochemical data of liquid films, modeling their properties becomes essential to investigate corrosion mechanisms. With different simulation approaches, corrosion rates are predicted and calibrated with experimental measurements. Numerical simulation offers new pathways for modeling atmospheric corrosion. Common methodologies include the finite element method (FEM), cellular automata (CA), and molecular dynamics (MD), providing different insights into the corrosion process. Their temporal and spatial scales are shown in Figure 12.

(1) The finite element method (FEM) involves macroscopic-scale analysis using continuum mechanics principles. (2) Molecular dynamics (MD) offers atomic-level modeling through Newtonian mechanics. (3) The Monte Carlo method comprises probabilistic statistical approaches at atomic/molecular scales. (4) Cellular automaton involves the discrete modeling of localized corrosion phenomena and corrosion dynamics simulation through evolution rules.

The accurate numerical modeling of liquid film physicochemical properties must address their inherent spatial heterogeneity and time-dependent behavior on material surfaces. For finite element simulations, this necessitates constructing a comprehensive three-dimensional distribution model of the liquid film metal corrosion system that integrates coupled physicochemical fields (electrochemical, thermal, and mass transport phenomena) with material-specific corrosion parameters. A precise understanding of the liquid film’s spatial distribution across the material surface forms the foundation for meaningful corrosion analysis.

For cellular automata simulations, the framework requires the precise definition of cellular state matrices and cellular state probabilistic transition rules. Similarly, molecular dynamics simulations demand focused attention on interatomic potential functions, which is the fundamental computational tools enabling the atomic-scale modeling of chemical corrosion processes.

### 4.1. Method Based on Finite Element Method (FEM)

#### 4.1.1. Principle

The finite element method (FEM) is a general-purpose numerical technique for solving partial differential equations. This approach divides complex systems into smaller elements represented as interconnected nodes. Through mathematical techniques such as variational principles or weighted residual methods, FEM transforms continuous differential equations into discrete algebraic formulations suitable for computational solution.

Metal corrosion is a complex electrochemical process driven by multi-phase interactions and coupled environmental factors. The core challenge of finite element simulations lies in the integration of corrosion kinetics and the mechanical behavior of liquid film corrosion and it employs multi-physics field coupling models. A typical FEM workflow involves constructing a three-dimensional geometric model of the system, defining material electrochemical and mechanical properties, applying actual boundary conditions (including mechanical loads and environmental conditions), and finally, coupling relevant physical fields such as electrochemical reactions, mass transportation, and stress distributions.

Common software for modeling liquid film corrosion includes COMSOL Multiphysics, CorrosionMaster, and BEASY. Among these, COMSOL Multiphysics is based on partial differential equation and widely adopted across scientific research and engineering applications. Its versatility lies in simulating coupled physical processes through robust multi-physics frameworks [44]. COMSOL Multiphysics has rich computational algorithms and allows modular add-ons for specialized applications.

CorrosionMaster is a simulation tool designed for corrosion risk assessment. Utilizing finite element analysis, it provides an advanced graphical modeling platform capable of generating high-precision 3D outputs. The software enables the precise evaluation of how different factors affect corrosion resistance, including material properties, protective coatings, structural designs, and environmental conditions.

BEASY, developed by Computational Mechanics (UK), is a corrosion simulation software employing boundary element methodology. The platform specializes in modeling and analyzing cathodic protection systems, electromagnetic field interactions (including underwater electric potential, and corrosion-related magnetic fields), and sacrificial anode corrosion dynamics. BEASY is widely applied in marine infrastructure and energy sectors, supporting corrosion control simulations for ships, offshore structures, and oil/gas storage and transportation systems [45].

#### 4.1.2. Application of Finite Element Method to Corrosion

The finite element method (FEM), as a differential equation-based numerical approach, enables physical field analysis across different 3D geometries, material compositions, and environmental conditions. Ruiz-Garcia, A. [46] has reviewed aluminum alloy corrosion models including uniform corrosion, galvanic corrosion, and localized corrosion phenomena, while also assessing the FEM’s role in corrosion simulation. Common corrosive environments include gas pipeline environment, soil environment, water environment, atmospheric environment, etc. In this paper, taking COMSOL as an example, we mainly focus on the atmospheric corrosive environment under the thin liquid film.

Atmospheric corrosion processes are influenced by multiple dynamic parameters, including fluctuating temperature, relative humidity, solar radiation, precipitation, wind, and pollutant concentrations [47]. These time-dependent environmental conditions interact synergistically to drive corrosion mechanisms and dynamics. There are two critical factors: the formation of thin liquid films on metal surfaces and the deposition of corrosive pollutants. Consequently, finite element simulations of atmospheric corrosion require precise modeling of the liquid film’s spatial-temporal physicochemical properties (distribution across the metal substrate and variation along with time). Due to the different distribution of liquid film on the material surface, corrosion on the material surface can be categorized into uniform corrosion on the surface and pitting corrosion on the point.

In uniform corrosion studies, Chen [48] employed finite element modeling to investigate galvanic corrosion between AerMet100 steel and Al7050 alloy under atmospheric conditions. By simulating current distribution across a 100 μm electrolyte layer, this work demonstrated the complex electrochemical behavior of thin liquid films while validating the shell current model’s effectiveness in predicting potential gradients.

For pitting corrosion, Cai [49] studies Q235 steel with defective passivation layers in marine environments and reveals how salt spray deposition accelerates passive layer rupture. Further investigations by Mollapour [50] combined experimental and numerical approaches to assess multi-factor pitting behavior. This research established nonlinear relationships between applied potential, exposure time, and pit depth evolution, confirming passivation film failure as the critical driver of pitting propagation. Zhu [51] developed a COMSOL-based finite element model to analyze corrosion layer evolution in reinforced concrete. By incorporating chromium and nickel elements into pitting simulations, this work elucidated how corrosion products of weathering steels can inhibit pit development through protective rust layer formation.

Extensive research on atmospheric corrosion at the material level has laid the groundwork for investigating more complex structural and environmental interactions, including galvanic corrosion between different metals, crevice-induced corrosion, stress corrosion, and corrosion fatigue mechanisms. In galvanic corrosion studies, Qingmiao [52] conducted finite element simulations of Al7050 aluminum alloy and AerMet100 steel, and revealed that increasing the cathode-to-anode area ratio accelerates aluminum dissolution without reversing electrode polarity. Palani, S. [53] modeled galvanic interactions between AA2024 aluminum and carbon fiber-reinforced polymers under thin electrolyte layers, and validated the prediction accuracy of current density and potential distribution. Galvanic corrosion principles also support anticorrosion coating design. Presuel-Moreno, F.J. [54] and Moraes, C.V. [55] demonstrated this on Al-Co-Ce alloy coatings and AA2024 Mg-rich printers, with their FEM framework accurately predicting corrosion potentials in AA2024 substrates.

In the crevice environment, Liu [56] analyzed the galvanic coupling corrosion between the crevices and found that the crevice width and thickness of the UNS A97050 plate had a significant effect on the potential and current distribution. A preliminary FEM model incorporating experimental electrochemical kinetics further revealed how boundary condition variations drive different corrosion behaviors. Cai [50] also detected the intergranular corrosion of Q235 steel in the marine atmosphere based on the COMSOL electrochemical module.

Under stress and cyclic loading conditions, the corrosion process is significantly accelerated. Haydar, S.A. [57] investigated this synergy by developing a computational model for atmospheric corrosion in the weld zones of an excavator arm under Mediterranean climate conditions. Using COMSOL Multiphysics, the study simulated the progression of a 2 mm diameter crack, revealing escalating stress concentrations in contact areas and continuous crack enlargement during corrosion propagation. Alsit, A. [58] simulated stress corrosion cracking in AISI 4340 steel specimens. The COMSOL-based predictions showed strong agreement with experimental results, including corrosion rate and stress-strain curve. Ding [59] employed COMSOL to quantify stress concentration factors across various corrosion pit geometries. The effects of environment factors (corrosion rate, corrosion pit morphology, and frequency of fatigue loading) on the fatigue life of steel wire were further analyzed.

These investigations span diverse corrosion types and material systems, employing varied experimental and computational approaches. Collective findings demonstrate strong consistency between finite element simulations and actual experimental data, validating numerical methods as reliable predictive tools. As an effective platform for simulating multi-physics corrosion processes, COMSOL Multiphysics can also reduce experimental time and resource expenditures.

### 4.2. Method Based on Cellular Automaton (CA)

#### 4.2.1. Principle

Cellular automaton is a type of method that is based on the discretization of time, space, and state, defining specific cellular evolution rules to reflect mesoscopic or atomic scale physical and chemical systems. Cellular automaton is a discrete dynamic model composed of finite state cells, where cells in space evolve according to local rules over a certain period of time.

The cellular automaton method mainly includes cellular states, cellular neighbors, and evolution rules. The cellular state represents the cellular position and attributes. Cellular neighbors are sets of surrounding cells. Common models of cellular automaton include the Von Neumann type, the Moore type, and its extended forms [60]. Local rules determine how cells evolve over time and are the core of model construction. By changing the definition of local rules, researchers have constructed cellular automaton models for simulating metal corrosion in different scenarios.

In 1983, Stephen Wolfram systematically introduced the theory and applications of CA, emphasizing its importance in complex system modeling [61]. With the advancement of the theory and computing power of cellular automaton, different types of cellular automaton are developed.

(1) **Multi-dimensional cellular automaton.** The initial cellular automaton is one-dimensional. In order to simulate more complex systems, cellular automaton is extended to two-dimensional and three-dimensional scenarios. Multi-dimensional cellular automaton can better simulate complex structures.

(2) **Probabilistic cellular automaton.** Probabilistic properties are introduced into cellular automaton, where state transitions are not deterministic but rather based on a certain probability distribution. The modified model can better describe and simulate processes and phenomena with strong randomness.

(3) **Heterogeneous cellular automaton.** Heterogeneous cellular automaton contains multiple types of cells. Their initial state is determined based on the spatial distribution of the material’s microstructure. Different cells follow different rules. This type of cellular automaton can be used to simulate systems with spatial heterogeneity.

(4) **Adaptive Cellular Automaton.** Traditional cellular automaton has fixed rules in space and time. In adaptive cellular automaton, the evolution rules for cells are dynamically updated based on their local environment or the system’s evolution pattern.

(5) **Continuous cellular automaton.** Continuous cellular automaton can model both continuous cell states and continuous time, making it particularly suitable for continuously evolving systems, such as those in fluid mechanics.

(6) **Other types of cellular automaton.** Quantum cellular automaton, machine learning-driven cellular automaton, dynamically topologized cellular automaton, hierarchical emergent cellular automaton, and asynchronously updated cellular automaton are also proposed. These approaches have demonstrated practical utility across various fields, such as quantum mechanics, artificial intelligence, network interactions, and biomedicine.

#### 4.2.2. Application of Cellular Automaton to Corrosion

In the field of metal corrosion kinetics simulation, researchers have achieved the accurate characterization of corrosion rate changes by constructing an evolution rule model based on corrosion mechanisms. Stafiej [62] established a two-dimensional cellular automaton model for metal corrosion and passivation problems. This model is based on the anode/cathode reaction of electrochemical corrosion and simulates the dynamic process of pitting damage initiation and non-planar damage evolution towards cavities after metal protective film rupture. Zenkri [63] further proposed a three-dimensional cellular automaton model, which integrates the coupling mechanisms of multiple physical fields such as electrochemical reactions, mass transfer, solution neutralization, and passivation characteristics of oxide layer. They found that the spontaneous spatial separation phenomenon in the anode/cathode region is significantly correlated with the degree of metal surface exposure and passivation state. Caprio’s team [64] studied the influence of grain orientation on corrosion pathways through a three-dimensional intergranular corrosion model system, confirming the evolution of metal surface roughness is strongly correlated with crystallographic properties and dissolution kinetics.

To capture the spatial difference and randomness of the actual corrosion process, heterogeneous cellular automaton and probabilistic cellular automaton are developed. Stephen Wolfram first systematically analyzed the emerging statistical behavior under deterministic rules in his work “Cellular Automaton and Complexity” (1983), providing a theoretical framework for the establishment of probabilistic models. On this basis, Vautrin El [65,66] and Taleb [67] developed a probabilistic cellular automaton based on the electrochemical diffusion mechanism. By embedding random events in the rules to characterize the actual reaction kinetics, they successfully reproduced the morphological evolution of corrosion pits. Di Caprio [68] introduced heterogeneous rules and constructed a corrosion model that includes passivation/de-passivation probabilistic parameters, quantitatively revealing the influence of passivation stability on the corrosion process. Saunier [69] simulated the dynamic growth process of corrosion product films at the metal/solution interface using a diffusion-controlled model.

The adaptivity and evolvability in cellular automata originated from research on the self-organization of complex systems. In 1993, Mitchell [70] investigated the self-reproduction and evolution capacity of adaptive cellular automaton. By optimizing cellular rules via genetic algorithms, the method can simulate complex structures and adapt to environmental changes. This work is recognized as one of the foundational contributions of adaptive cellular automaton.

Research on adaptive and evolutionary cellular automata continues to yield new breakthroughs. Hanna Derets [71] proposed an adaptive and evolutionary cellular automaton model based on genetic algorithms to simulate Darwin’s rules in biological evolution. The model dynamically adjusts local survival conditions to simulate the survival status of populations and biological evolution under changing natural environmental conditions.

Continuous cellular automaton was first proposed in 2009 by Kazumasa Tomita [72] to overcome the limitation of discrete values in modeling complex systems and their behaviors. This method is based on differential equations and extends cell states to continuous real numbers. It has widespread application in various fields, such as metallic corrosion, fluid dynamics, computer technology, and artificial intelligence.

Puspa Eosina [73] applied continuous cellular automata to study the mechanisms of viral transmission and spread, modeling community neighborhoods as individual continuous cells. By leveraging the mechanisms of infectious disease transmission, they proposed a novel continuous cellular automata-based model for the spread of the novel coronavirus and obtained precise predictions of the virus’s transmission range and trends.

Cellular automata have achieved various advances in corrosion simulation. Probabilistic, heterogeneous, adaptive, and continuous frameworks have been proposed. They are able to capture the complexity of the corrosion systems, including continuous electrochemical processes, microstructural heterogeneity, stochastic uncertainty, and dynamic variations. However, challenges still persist and new breakthroughs are needed.

First, the interpretability of complex rules is questionable. It is difficult to correlate model parameters with corrosion processes. Second, computation complexity increases exponentially when coupling multi-physics fields (i.e., electrochemical reactions, mass transport, stress fields). High-resolution simulations are limited. Third, there are no standard experimental validation frameworks. Parameter calibration and error analysis cannot be achieved. Machine learning-driven self-optimization, meso–macro unified modeling, and establishing comprehensive experimental validation frameworks are promising solutions.

### 4.3. Method Based on Molecular Dynamics (MD)

#### 4.3.1. Principle

Molecular dynamics is based on two fundamental hypotheses: (1) Particle motion follows Newton’s laws of motion; and (2) interparticle forces adhere to the principle of superposition. By applying Newton’s laws, the method calculates atomic trajectories over time by deriving accelerations from net forces acting on each atom, then iteratively solving equations of motion to update positions and velocities.

A typical molecular dynamics workflow involves (1) defining the simulated system (atomic species, quantity, initial coordinates, and velocities, etc.), (2) applying boundary conditions (periodic, fixed, free, or other boundary conditions), (3) calculating interatomic forces (covalent bonds, electrostatic interactions, and van der Waals forces, etc.), (4) solving equations of motion and updating particles’ state, (5) obtaining data from the simulation and analyzing the particle motion characteristics, and (6) visualization and post-processing.

Classical molecular dynamics simulations offer a robust framework for probing the behavior of complex systems and elucidating the potential physical mechanisms. These simulations bridge atomic-scale interactions to macroscopic phenomena, enabling precise predictions of material properties, chemical reaction pathways, and biomolecular dynamics. For molecular dynamics (MD), LAMMPS [74] and Materials Studio (MS) [75] are the most commonly used platforms.

LAMMPS is a compiled software package designed for large-scale complex system simulations, while Materials Studio (MS) serves as a user-oriented interactive platform better suited for rapid analysis of relatively smaller systems.

#### 4.3.2. Application of Molecular Dynamics to Corrosion

Andersen [76] developed the control methods for temperature and pressure in molecular dynamics and established numerical simulation methods for constant temperature and pressure. Early applications of MD simulations characterized corrosion through atomic diffusion and adsorption behaviors. Selecting appropriate interatomic potential functions is the key for the MD simulaitons.

The diffusion phenomena of Fe in liquid metals were studied by Arkundato [77] using the simplest Lennard-Jones (LJ) potential function.

The diffusion coefficients of Fe and Ni in liquid Pb-Bi eutectic were calculated by Gao [78] using the embedded atom (EAM) potential function to study the corrosion behavior of stainless steel.

The Reactive Force Field (ReaxFF) method effectively captures the interatomic interactions (bond breaking and formation) through its bond order (BO). The bond order value BO varies continuously with parameters such as interatomic distance and electronegativity difference. Bonds break when BO approaches zero, while new bonds form when BO exceeds zero. This approach overcomes the limitation of traditional fixed-bond force fields in describing chemical reaction processes.

The energy of the system consists of multiple terms and can be expressed as

(3) $$E_{SYSTEM}=E_{BOND}+E_{OVER}+E_{ANGLE}+E_{TORS}+E_{VDW}+E_{COU}+E_{SPECIFIC}$$

where $E_{BOND}$ is the bond energy term, $E_{OVER}$ is the energy correction term for over-coordination, $E_{ANGLE}$ is the valence angle energy term, $E_{TORS}$ is the torsion angle energy term, $E_{VDW}$ is the van der Waals interaction term, $E_{COU}$ is the coulombic interaction term, and $E_{SPECIFIC}$ is the system specific term. This energy function provides a unified description of both bonded and non-bonded states. Through synchronized dynamic updates of bond orders, atomic charges, and energy terms, it enables simulations of large-scale reactions.

ReaxFF force fields have gained significant traction in corrosion research, particularly for iron-based systems. DorMohammadi, H. [79] employed ReaxFF-MD to simulate the initial stage of Fe corrosion in pure water under different electric fields and temperatures (300–350 K). Farzi, N. [80] utilized ReaxFF molecular dynamics to investigate temperature-dependent (298 K and 500 K) passivation mechanisms of Fe_3_C in water and HCl solution. They also studied the influence of inhibitors on Fe_3_C corrosion in H_2_SO_4_ environments [81].

Qiu, Y. [82] employed ReaxFF molecular dynamics to study the corrosion of chlorine, hydrogen chloride, and water on iron covered with intact/damaged oxide film under 1300 K. The simulation results demonstrated the decisive role of the oxide film in alleviating corrosion. Du, L. [83] investigated carbon steel corrosion and found deep oxidation (due to lattice defect caused by Fe atom separation) and decarburization phenomenoa.

Thijs, L.C. [84] simulated liquid Fe-O systems at 2000 K through molecular dynamics using six different ReaxFF parameter sets and the simulation results showed significant differences. This finding suggests that ReaxFF needs to be improved to better capture the properties of liquid Fe-O systems. Kim [85] employed molecular dynamics to compare high-temperature corrosion behavior between Fe-Cr-Al alloys and pure iron. Simulations revealed that the segregation of alloying elements (Al and Cr) toward the surface produces lower stress gradients at the metal–environment interface relative to pure iron systems.

In terms of corrosion inhibition, Kumar [86] combined Density Functional Theory (DFT) and ReaxFF to investigate the synergistic inhibition mechanism of two inhibitors for copper corrosion. To better explore the mechanism of corrosion inhibitors, Ait Mansour, A. [87,88,89,90] took N80-CS as the base, employed different inhibitors, and combined molecular dynamics simulations and DFT calculations. They found that the corrosion inhibition effect of the inhibitor is mainly reflected in the adsorption behavior and the formation of covalent bonds. Kadhim, A. [91] reviews common corrosion inhibitors and their corresponding inhibition rates, while Samuel, H.S. [92] reviews the technical methods that will be used in corrosion inhibition. This approach provides a useful method for screening high-performance inhibitor combinations in industrial applications.

In summary, molecular dynamics simulations now enable the study of metal corrosion across various environmental conditions, including atmospheric, marine, room, and high-temperature environments, and so on. By modeling atomic-scale interactions, MD analyzes the motion patterns, energy, and structural changes of metal atoms, which can provide an atomic viewpoint for investigating the internal mechanism of the corrosion process.

### 4.4. Current Challenges and Opportunities

**Figure 13:**
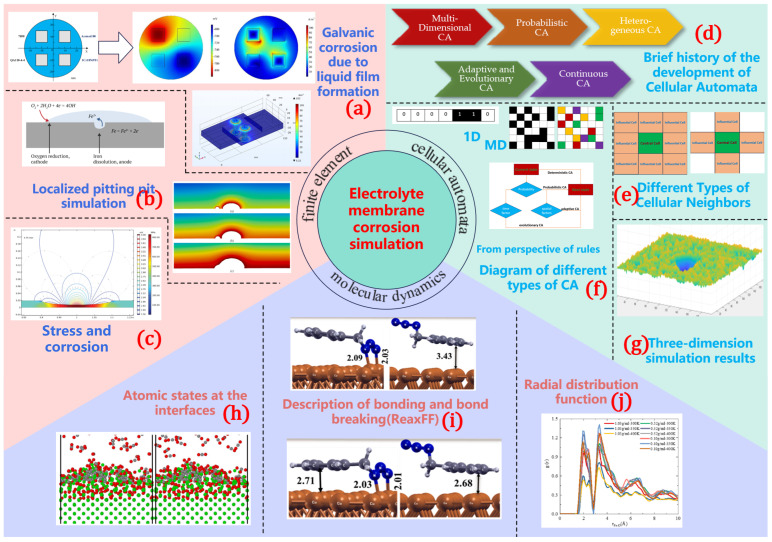
Functionalities of FEM, CA, and MD simulation methods. (**a**) Galvanic corrosion due to liquid film formation [49]; (**b**) Localized pitting pit simulation [59]; (**c**) Stress and corrosion; (**d**) Brief history of the development of Cellular Automata; (**e**) Different Types of Cellular Neighbors; (**f**) Diagram of different types of CA; (**g**) Three-dimension simulation results; (**h**) Atomic states at the interfaces; (**i**) Description of bonding and bond breaking(ReaxFF) [86]; (**j**) Radial distribution function [84].

The three simulation methods each have their own characteristics (in Table 4) as well as applications (in Figure 13).

The three methods can be used in combination to balance and bridge the macroscopic performance and microscopic mechanisms. For example, in order to overcome the limitations of individual simulation method in terms of time and space scales, MD and FEM can be combined. This combination enables the description of micro-scale corrosion mechanisms, the capture of macro-scale corrosion patterns, and the unification of cross-scale parameters. The coupling simulation of FEM and MD enables multi-scale computation bridging microscopic and macroscopic domains.

**Figure 14:**
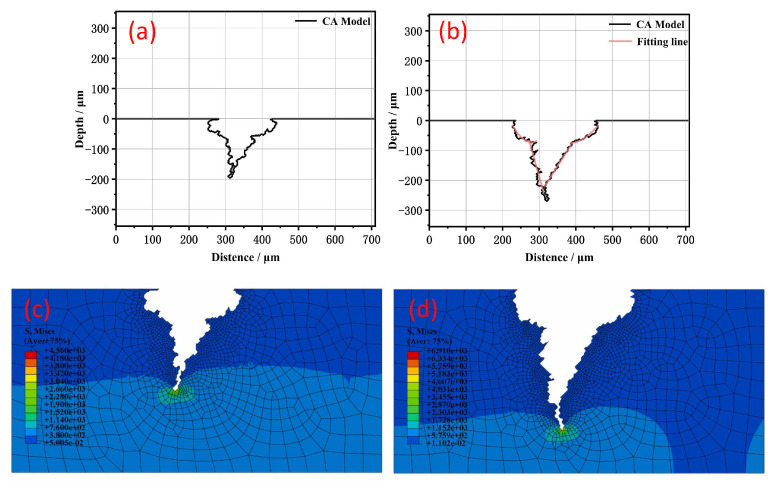
(**a**,**b**) The evolution process of the cross-sectional stress corrosion profiles (using cellular automaton); (**c**,**d**) stress distribution states of cross-sectional stress corrosion cracking profiles at different stages of corrosion (using finite element method) [93].

Taking the combination of FEM and CA as an example, Gong, K. [93] used cellular automata and finite element analysis to model the stress corrosion cracking process of pipeline steel and simulated the crack initiation and propagation. Some of the results can be seen in Figure 14. The combination of the two tools can comprehensively analyze the influencing factors such as hydrogen diffusion, corrosion product deposition, metal oxidation rate, etc.

These simulation methods may provide a clearer understanding of corrosion mechanisms, enable the development of reliable corrosion prediction models, and have broader application prospect in various regions. In the field of liquid film corrosion, the combination of simulation methods needs further research, which is also one of the focuses of our future work.

## 5. Conclusions

Atmospheric corrosion under thin liquid film represents a critical failure mechanism for industrial equipment. This review mainly addresses two aspects in this field:

In terms of thickness measurement, the electrical measurement method and microwave, ultrasonic, and optical interferometry are the most commonly used methods. For chemical composition measurement, common methods include titration analysis, ion-selective electrodes, and X-ray fluorescence (XRF). For conductivity measurement, the AC bridge method and the multi-electrode method are widely used. As the liquid film is non-continuous and non-uniform on the metal surface and varies along with time, it is challenging to measure its properties. Among all these measurement methods, white light interferometry enables high-precision in situ thickness measurements without damaging the liquid film. X-ray fluorescence (XRF) provides in situ, non-contact elemental analysis for ion concentration. For conductivity measurement, the four-electrode system has a high measurement accuracy of intrinsic resistance and is not affected by polarization and contact resistance.

After obtaining the properties of the liquid film, corrosion simulations can be conducted. Different simulation methods have their own strengths. The finite element method (FEM) is a powerful tool for macro-scale analysis. Molecular dynamics (MD) simulations are good at investigating the atomic-scale corrosion mechanisms. Cellular automaton (CA) models are particularly suitable for simulating the evolution of long-term corrosion process. The combination of multiple simulation methods is a promising way to invesigate corrosion mechanisms at the micro-scale and predict corrosion behavior at the macro-scale.

## Figures and Tables

**Table 1:** Comparison of different thickness measurement methods.

Methods	Advantages	Limitations
Electrical measurement method	High time resolution, simple to operate, and convenient for data processing.	Destroying the shape of liquid film, poor effect on non-conductive liquid film, and low spatial resolution (>100 μm). The accuracy depends on the equipment of measuring displacement.
Microwave method	Non-contact measurement, high reliability, and suitable for dynamic monitoring.	The precision is low (about 100 μm), which is limited by wavelength of microwave.
Ultrasonic method	Sensitive to multi-layer media and can measure the thickness of liquid film with rigid boundary.	The signal-to-noise ratio of air-liquid interface is low and the measurement is complex. The precision is about 10 μm.
Optical method	High precision (such as nanometer interference method), non-contact, and strong stability.	The measurement range of polarization method is narrow, and the white light interferometry needs complex calibration. The precision is about 0.1 μm.
Fluorescence intensity method	High signal-to-noise ratio and convenient for global brightness analysis.	Fluorescent agents need to be added so it will interfere with the original components of the liquid film. The precision is about 100 μm.

**Table 2:** Average value of experimental data and error [33].

Experimental Parameter	Intracellular pH	Membrane Potential	Intracellular $H^{+}$ Concentration	ΔpH $\left(10\%{\mathrm{CO}}_{2}\right)$	ΔpH $\left(5\%{\mathrm{CO}}_{2}\right)$
Mean Value	7.41	44.2 mV	40.1 nM	0.58 unit	0.42 unit
Standard Error	±0.015	±0.76	±1.3	±0.02	±0.01

**Table 3:** Comparison of different composition measurement methods.

Parameters	Methods	Advantages	Limitations
Chloride ion concentration	Mohr Titration Method	Operate easily rapidly	Low accuracy with interfering ions
	Ion-Selective Electrode (ISE) Method	Fast and efficient, suitable for strong acid/alkaline solutions	The electrode may interfere with the pH value
	X-ray Fluorescence (XRF) Analysis Method	Non-destructive, high-resolution, supporting multi-element analysis	High cost, weak anti-interference ability, essential laboratory environment
	Microwave Detection Method	Non-invasive, fast response, suitable for complex solutions	Accuracy is easily affected by solution properties, resulting in high equipment cost
pH value	Glass Electrodes	Mature technology, stable response	Susceptible to ion interference, requiring regular calibration
	Surface-Enhanced Raman Spectroscopy (SERS)	Unlabeled, high sensitivity, and capable of real-time monitoring	Complex preparation in substrate surface and high cost
Conductivity	AC Bridge Method	Good stability and high accuracy	Complex operation and long measurement time
	Three-Electrode Method	Precise control of potential, separation of interface and bulk response	Unable to directly measure the conductivity of the body, the system is complex, and there is interference from solution resistance
	Four-Electrode Method	It can directly measure the conductivity of the body, completely eliminate polarization errors, and has simple and fast operation	Unable to analyze interface characteristics, requires precise geometric constants, and has limitations in high-frequency measurements

**Table 4:** Comparison of different simulation methods. (The bolded part is a general description of their characteristics.)

Principle	FEM	Discretize complex systems into small units and solve partial differential equations.
	CA	Simulates the dynamic behavior of corrosion processes based on discrete space-time and local evolution rules.
	MD	Simulates atomic movement by solving Newton’s equations of motion using interatomic forces.
Advantages	FEM	**High accuracy:** Captures details such as local stress concentration and material performance degradation. **Wide applicability:** Can handle complex geometric structures and nonlinear problems. **Multi-field coordination:** Coupled fields simulation represents actual working conditions.
	CA	**Wide time range:** Suitable for corrosion evolution simulations over long time ranges. **Highly visualizable:** Dynamic changes in corrosion morphology are visually presented. **Probabilistic simulation:** Reflects the non-uniformity and uncertainty of the corrosion process through probabilistic rules.
	MD	**Reproducing mechanisms:** Deep exploration into the microscopic mechanisms of corrosion. **Multi-environment applicability:** Supports corrosion research under extreme conditions (such as high temperature, high pressure, and supercritical media).
Limitations	FEM	**Insufficient microscopic mechanism:** Unable to investigate the corrosion mechanism at the atomic/molecular level.
	CA	**Parameter sensitivity:** The evolution rules and neighbor models have significant impacts on the results.
	MD	**Small spatial and temporal coverage:** Suitable for short time (nanosecond to microsecond) and microscopic (atomic scale) processes.
Input and output	FEM	**Input:** Geometric model of metal and liquid film, liquid film thickness, ion concentrations, conductivity, diffusion coefficient, temperature, etc. **Output:** Corrosion rate, corrosion current density, etc.
	CA	**Input:** Cell state set of metal and liquid film, cell state transition rules. **Output:** Corrosion morphology, probability distribution of corrosion parameters (corrosion loss, rate, etc.).
	MD	**Input:** Particle model of metal and liquid film, potential function. **Output:** Corrosion rate, product type and content.

## Data Availability

No new data were created or analyzed in this study. Data sharing is not applicable to this article.
